# Small intestinal angiosarcoma masquerading as an appendiceal abscess

**DOI:** 10.1308/003588413X13511609955373

**Published:** 2013-01

**Authors:** DSH Liu, H Smith, MMW Lee, M Djeric

**Affiliations:** ^1^Austin Hospital, Heidelberg, VIC,Australia; ^2^Royal Hobart Hospital, TAS,Australia; ^3^Royal Melbourne Hospital, VIC,Australia

**Keywords:** Angiosarcoma, Gastrointestinal, Tumour, Appendix, Abscess

## Abstract

Angiosarcomas of the small intestine are rare and present non-specifically. They usually manifest with abdominal discomfort, altered bowel habits, anaemia and gastrointestinal bleeding. Diagnosis is often challenging and occurs at an advanced tumour stage. We describe a case of a terminal ileum angiosarcoma masquerading as an appendiceal abscess, and discuss salient clinicopathological features in diagnosing and managing this disease.

Intestinal angiosarcomas are rare high grade vascular neoplasms.^1–3^ Diagnosis is challenging because of non-specific clinical, radiological and histopathological findings. We describe a case of a terminal ileum angiosarcoma masquerading as an appendiceal abscess and discuss salient clinicopathological features in managing this disease.

## Case history

**Figure 1:**
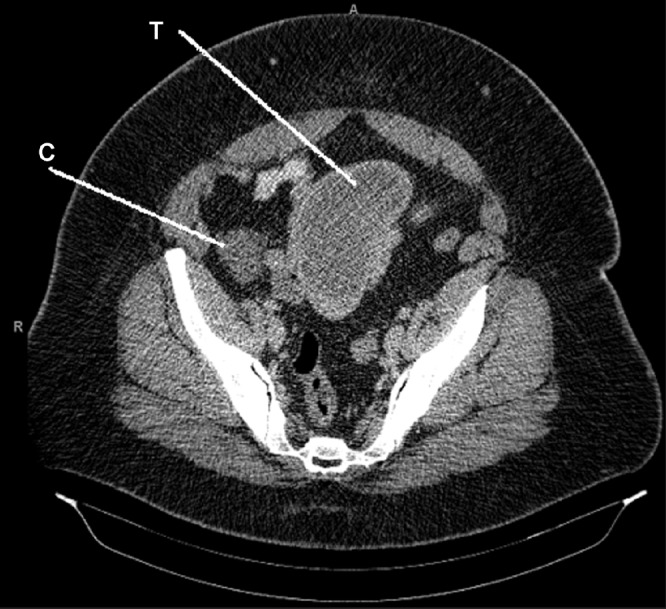
Axial computed tomography of the abdomen demonstrating a tumour (T) in the terminal ileum, which appears thick walled, located close to the caecal pole (C), mimicking an appendiceal abscess

A 39-year-old woman presented with three weeks of increasing right iliac fossa pain, abdominal bloating and vomiting. She denied previous abdominal surgery, radiation or industrial chemical exposure. She was febrile (37.9°C) and tachycardic (110bpm) on examination, and her abdomen was distended with tenderness and guarding localised to the right iliac fossa. Full blood examination revealed microcytic anaemia (haemoglobin 73g/l, mean corpuscular volume 77fl) and leukocytosis (white cell count 13.1 × 10^9^/l). C-reactive protein was elevated at 102mg/l. Abdominal computed tomography (CT) demonstrated a 10cm × 6cm × 4cm thick-walled mass adjacent to the caecal pole with a proximally dilated small bowel (Fig 1). No lymphadenopathy or other solid viscus abnormalities were detected. The uterus, Fallopian tubes and ovaries appeared unremarkable on pelvic ultrasonography.

**Figure 2:**
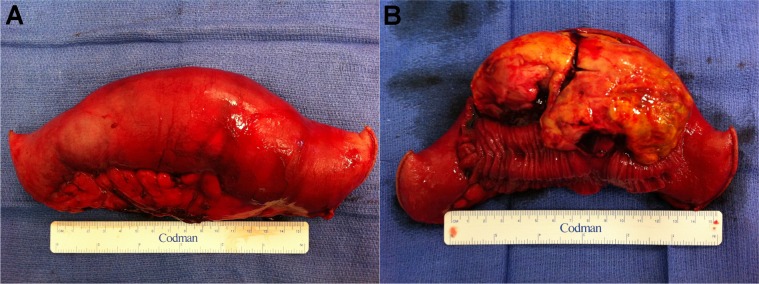
Segmental small bowel resection (A) with intestinal wall opened longitudinally along the antimesenteric border (B) to reveal a large mural angiosarcoma

The clinical picture was thought to be consistent with an appendiceal abscess and associated small bowel obstruction. Although CT guided percutaneous drainage of the abscess was proposed, it was decided that an exploratory laparotomy was more appropriate given the increased risk of bowel injury caused by navigating a wide bore needle through the abdominal cavity with distended bowel. On exploration, the appendix appeared uninflamed. However, a large obstructing mural tumour arising from the terminal ileum was identified. Consequently, a 17cm segment of ileum containing the tumour was resected (Fig 2) and a stapled anastomosis was performed.

**Figure 3:**
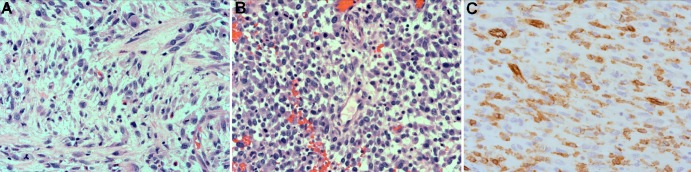
Haematoxylin and eosin stain of angiosarcoma demonstrating cellular pleomorphism with spindled and epithelioid shaped cells (A), primitive vessel formation (B) and positive cell membrane staining for the endothelial marker CD31 (C); 200× magnification

Microscopic examination of the resected specimen revealed an infiltrative malignancy involving the bowel wall and mesentery with evidence of venous invasion. Tumour morphology was suggestive of high grade sarcoma (Figs 3a and b), and consisted of cellular pleomorphism with mixed sheets of spindled and epithelioid cells, extensive areas of necrosis and cystic degeneration, cytoplasmic vacuolisation and intercellular vasoformation. Subsequent immunohistochemistry demonstrated a diffuse, strong positive reaction to endothelial markers (CD31 and CD34), and negative staining for stromal (CD117 and DOG1), smooth muscle (desmin), epithelial (MNF116), melanocytic (melan-A, HMB-45) and neural (S100) tissue markers, which together established the diagnosis of an angiosarcoma (Fig 3c). The patient’s postoperative recovery was uneventful with return of normal bowel function. She was referred to the Peter MacCallum Cancer Centre in Victoria, Australia, for consideration of adjuvant chemotherapy and ongoing specialist sarcoma management.

## Discussion

Angiosarcomas are high grade vascular neoplasms that account for 1–2% of all soft tissue sarcomas.^1^ These tumours have a predilection for skin and subcutaneous tissues,^1,2^ and less commonly manifest in the breast, liver, spleen, bones, ovaries and adrenal glands.^2^ Intestinal angiosarcomas are exceedingly rare. In an extensive review of the English literature since 1970 by Grewal *et al*,^2^ fewer than 30 cases have been reported, of which only 4 were found in the terminal ileum.

The clinical presentation of intestinal angiosarcoma is non-specific. Common symptoms include abdominal discomfort, nausea, vomiting and altered bowel habits.^1,2^ The presence of unexplained anaemia, gastrointestinal bleeding and bowel obstruction may increase suspicion for an underlying malignancy.^2,3^ While the pathogenesis of intestinal angiosarcoma is unclear, several pathogenic associations have been identified. These include: chronic lymphoedema; previous irradiation; exposure to industrial chemicals such as thorotrast, vinyl chloride and arsenic; long-term peritoneal dialysis; persistent intra-abdominal foreign body; and visceral metastasis from Kaposi’s sarcoma.^2–4^

Due to the anatomical location of the tumour, CT has limited diagnostic utility. Occasionally, direct tumour visualisation can be achieved endoscopically. However, conventional endoscopy is only useful for tumours located in the stomach, duodenum and colon. For jejunum and ileum tumours, some authors have advocated the use of capsule endoscopy and barium studies but with limited diagnostic success.^5^ Exploratory laparotomy is often required to reach a diagnosis.^2^

Our report of a case of intestinal angiosarcoma masquerading as an appendiceal abscess reiterates the variable nature in which this tumour may manifest. Our patient had no history of lymphoedema, toxic exposure or radiation therapy. Her three-week history of right iliac fossa pain, systemic symptoms and signs of sepsis, with localised guarding, and CT evidence of a thick-walled collection adjacent to the caecum supported the provisional diagnosis of an appendiceal abscess. However, this would not explain her microcytic anaemia.

Establishing the correct diagnosis in this case was of paramount importance because the management approach, disease progression and prognosis differs vastly between a tumour and an abscess. The initial management of an appendiceal abscess is usually conservative with percutaneous drainage and intravenous antibiotics, followed by a limited right hemicolectomy if the sepsis does not resolve. The outcome of an appendiceal abscess is typically favourable with rapid recovery of the patient. In contrast, early surgical resection and adjuvant chemotherapy are the definitive treatments for angiosarcomas. Unfortunately, despite this, angiosarcomas are universally fatal with an overall survival of less than one year.^2,3^ Furthermore, attempts at percutaneous drainage may be complicated by occult bowel injury, delayed diagnosis and treatment, and potential tumour seeding along needle tracts.

The pathological identification of an intestinal angiosarcoma can be challenging. Morphologically, it may appear similar to a leiomyosarcoma, a gastrointestinal stromal tumour, a metastatic melanoma, a lymphoma or a poorly differentiated carcinoma. Immunohistological profiling is mandatory to achieve a diagnosis.^2^ The classic microscopic appearance of an intestinal angiosarcoma consists of a network of anastomosing, delicate vascular channels lined by atypical endothelial cells mixed with solid sheets of spindled, epithelioid or undifferentiated cells. Frequently, there are areas of cystic degeneration and cellular necrosis.^1^ Immunohistologically, intestinal angiosarcomas are typically positive for endothelial markers (eg CD31 and CD34) and negative for epithelial markers (eg keratin).^1,2^ In our case, the morphological and immunohistological findings were consistent with an angiosarcoma and not neural, melanocytic, epithelial, smooth muscle or stromal in origin.

## Conclusions

Even though intestinal angiosarcomas are rare, they should be considered when protracted abdominal pain and bowel obstruction presents in the setting of unexplained anaemia or gastrointestinal bleeding, especially in the absence of previous abdominal surgery. Immunohistochemistry is mandatory to establish a diagnosis of angiosarcoma.
